# A Distributed Energy-Balanced Topology Control Algorithm Based on a Noncooperative Game for Wireless Sensor Networks

**DOI:** 10.3390/s18124454

**Published:** 2018-12-16

**Authors:** Yongwen Du, Junhui Gong, Zhangmin Wang, Ning Xu

**Affiliations:** School of Electronics & Information Engineering, Lanzhou Jiaotong University, Lanzhou 730070, China; 0217603@stu.lzjtu.edu.cn (J.G.); 0217596@stu.lzjtu.edu.cn (Z.W.); 0616321@stu.lzjtu.edu.cn (N.X.)

**Keywords:** wireless sensor networks, topology control, game theory, energy balanced

## Abstract

In wireless sensor networks, nodes may adopt selfish behavior to save their energy resources, which causes energy imbalance among nodes, because of lacking a central controller with the function of making nodes cooperate. Noncooperative game is an effective tool for portraying this kind of selfish behavior. In this paper, we address the problems of transmission power minimization and energy balance using a topology control game. Firstly, we establish a topology control game model and prove that the topology game model is an ordinal potential game with Pareto optimality. Secondly, based on this model, we propose an Energy Balance Topology control Game algorithm (EBTG), in which, by taking the energy efficiency and energy balance of the nodes into account, we design an improved optimization-integrated utility function by introducing the Theil index. Finally, simulation results show that the EBTG algorithm can improve the energy balance and energy efficiency, and can prolong the network lifetime in comparison with other topology control algorithms based on game theory.

## 1. Introduction

WSN (Wireless sensor networks) usually consist of a large number of sensor nodes with which we can sense and collect external environmental information. In WSN applications, in views of limited power resource (batteries) and long lifetime requirement, the energy consumption becomes the core issue in WSN research [1]. Topology control is one of the most important considerations for network life. The goal of topology control is to optimize the transmitting power of each node and construct a better topology to improve network performance and prolong network lifetime [2].

At present, in the field of wireless sensor networks, many topological control algorithms, which are mainly divided into hierarchical, power control, and game-type topology control algorithms, are proposed. Kang et al. [3] proposed a low-power hierarchical WSN topology control algorithm, which is a multilevel topology control algorithm; this algorithm extends the network level and improves the maintainability of WSN using a combination of the static address and the dynamic address. In the work of [4], an energy-efficient hierarchical topology control method is established in WSN using time slots, in which a cluster-head selecting approach decreases the difference in the cluster size of LEACH and the responsibility mechanism for the active node makes the energy consumption uniform in the cluster. Kubisch et al. [5] implemented dynamic power control to set the node degree of the upper and lower limits, thus resulting in a lower total energy consumption network topology. The power control algorithm proposed in [6] uses a Borel Cayley graph to construct a network topology that has a short average link and low energy consumption. The algorithm does not consider the robustness of the network topology and the residual energy of nodes, which affects the operation of the network to some extent.

When the sensor nodes perform data forwarding, the node will show selfish behavior due to energy saving considerations, and competition will occur between nodes [7]. On this basis, the game theory approach can be introduced into the study of WSN topology control. Game theory provides a powerful tool [8] for describing the phenomena of competition and individual coping strategies between intelligent rational decision-makers, and it has been used in systems concerning action and payoff. Komali et al. [9,10] formulated energy-efficient topology control as a noncooperative potential game, which guarantees the existence of at least one Nash equilibrium (NE) and proposes a distributed noncooperative game topology control algorithm based on game theory. Max-improvement algorithm (MIA) and δ-improvement algorithm (DIA) are proposed to adjust the per-node power-level so that the resulting topology is energy efficient and this can satisfy current global properties such as strong connectivity. In the work of [11], a topology control algorithm based on a link power consumption game is designed to run the minimum MLPT algorithm for the maximum power of the node; the algorithm defines a new TC game, in which nodes are able to dynamically adjust their transmission power in a per-packet manner, and try to minimize their energy usage through considering both traffic load and transmission power parameters. To consider network lifetime as well, researchers have proposed two game-based topology control algorithms, namely the virtual game-based energy-balanced algorithm (VGEB) [12] and the energy welfare topology control algorithm (EWTC) [13], which have been developed to improve network lifetime via energy-balanced network topologies. The VGEB algorithm greatly minimizes the energy exhaustion in the information exchange and considers the energy balance that can maximize the network lifetime. The EWTC algorithm proposed to select the power levels of nodes according to the trade-off between power level and energy population, quantified by the welfare metric. In the EWTC algorithm, each node makes decisions to maximize the energy welfare of its local society. In the work of [14], the adaptive cooperative topological control algorithm (CTCA) based on game theory considers the smallest potential lifetime and degree as the primary and secondary utility functions. The CTCA algorithm reduces the total energy consumption of network and the difference in energy between nodes compared with other topology control algorithms based on game theory. In the work of [15], a topology control algorithm (DEBA) based on the ordinal potential game is proposed by designing a payoff function that considers both network connectivity and the energy balance of nodes; the proposed algorithm has fewer bottleneck nodes that feature heavy traffic load and low residual energy, and smaller variance of node residual energy, thus achieving a longer life. Although some of the above mentioned algorithms based on game theory can achieve network topology control and improve network performance, they cannot guarantee the connectivity and robustness of the network. Additionally, the remaining energy, energy balance and energy efficiency of the nodes are not fully and accurately considered.

Based on the foregoing analysis, in this paper, we consider the residual energy, transmission power and degree of the node for the energy efficiency and energy balance of the nodes in the network. In addition, an Energy Balance Topology control Game algorithm (EBTG) is proposed which employs the improved optimization-integrated utility function by introducing the Theil index. The network topology constructed by this algorithm can guarantee the connectivity and robustness of the network and balance the energy between nodes, and also can effectively prolong the network lifetime.

The rest of the paper is organized as follows. Section 2 reviews critical concepts of the network model and the theory of potential games as applicable to our problem. Section 3 presents the topology control game model and provides the game formulation and theoretic analysis. From this model, in Section 4, an energy-balanced topology control algorithm, in which each sensor adaptively adjusts its transmit power according to the residual energy, is proposed. Section 5 validates our EBTG algorithm via simulation. Finally, Section 6 concludes this paper.

## 2. Preliminaries

In this section, we present a brief overview of some fundamental concepts related to the network model, the ordinal potential game theory and the Theil index.

### 2.1. Network Model

WSN are usually abstracted G=(N,L,P) according to graph theory. Let G=(N,L,P) be an undirected graph, where *N* denotes the set of nodes, *L* is a set of two-node communication links in node set *N* at time *t*, and *P* represents the transmit power set of *n* nodes.

It is assumed that all nodes are randomly deployed in the plane monitoring area. We only consider multi-hop network environments for wireless sensor network. We assume that every node is capable of adjusting its transmission power dynamically, i.e., a node can use a different power to reach each of its neighbors. The outcome of research on MAC-layer protocols [16] provides the feasibility of such an assumption. When the transmit power $p_{i}\in \left[0,p_{i}^{max}\right]$ of node *i* is sufficiently large, the signal received by node *j* is higher than the receiving threshold *p* so that node *j* can respond.

Because most routing and channel studies use bidirectional links, it is assumed that the links in the network topology are bidirectional. Link bidirectionality is also crucial for proper functioning of MAC protocols such as 802.11 [17]. Hence, we assume that links in our network model must be bidirectional to be useful. When all nodes use their maximum power to communicate, the formed network topology is denoted by $G_{max}$. In this design, $G_{max}$ is the connected network.

### 2.2. Ordinal Potential Game Theory

The ordinal potential game is a kind of strategy game. The strategy game Γ consists of *N* players, the possible strategy *S* of the players, and consequences *u* of the strategy. The following definition is given for the strategy game:

(1) $$\Gamma =\langle N,S,\left\{u_{i}\right\}\rangle$$

Three definitions are as follows: (1) N={1,2,3,⋯,n} represents the set of players, and *n* is the number of players in the game. (2) *S* represents the policy space, and *S* is the Cartesian product of the set of policies $S_{i}\left(i\in N\right)$, where $S_{i}=\left\{s_{i1},s_{i2},\cdots ,s_{ik}\right\}$ represents an optional set of policies for node *i*, which is usually abbreviated as $S_{i}=\left\{s_{1},s_{2},\cdots ,s_{k}\right\}$. In general, we use $s=(s_{i},s_{-i})\in S$ to describe a strategy combination, $s_{i}$ to represent the strategy choice of node *i*, and $s_{-i}$ to represent the other node strategy choices except node *i*. (3) *u* represents the utility function $u=\{u_{1},u_{2},\cdots ,u_{n}\}$, where $u_{i}^{max}$ denotes the maximum utility function that node *i* can achieve in the policy combination $\left(s_{i},s_{-i}\right)$.

**Definition** **1.** A strategy tuple $s^{*}=\left\{s_{1}^{*},\cdots ,s_{n}^{*}\right\}$ is a Nash Equilibrium (NE) [18], if $s_{i}^{*}$ is the best response to $s_{-i}^{*}$ for every player i. Formally, the strategy tuple $s^{*}$ is an NE, if $u_{i}\left(s^{*}\right)\geq u_{i}\left(s_{i},s_{-i}^{*}\right)$, for $\forall i\in N,\forall s_{i}\in S_{i}$.

A game may possess a large amount of NEs or none at all. Thus, it is of interest to design the utility function in a way such that the game has at least one NE point. Fortunately, a kind of games called potential games with compact strategy spaces are known to possess at least one NE [19].

**Definition** **2.** *A strategic game $\Gamma =\langle N,S,\left\{u_{i}\right\}\rangle$ is an ordinal potential game if there exists a function V such that ∀i∈N, $\forall s_{-i}\in S_{-i}$ and for $\forall a_{i},b_{i}\in$$S_{i}$*

(2) $$V\left(a_{i},s_{-i}\right)-V\left(b_{i},s_{-i}\right)>0\Leftrightarrow u_{i}\left(a_{i},s_{-i}\right)-u_{i}\left(b_{i},s_{-i}\right)>0$$

*The function V is called the ordinal potential function of the strategy game* **Γ** . Then, the strategy combination $s^{*}$ for the maximum value of the ordinal potential function V is the NE of the game [19].

### 2.3. Theil Index

The Theil index [20] is a statistic primarily used to measure economic inequality and other economic phenomena. It was proposed by econometrician Henri Theil at Erasmus University in Rotterdam. This index measures income inequality through the concept of entropy in information theory [21]. Whereas the concept of the entropy index in information theory is used to measure the income gap, the income gap can be interpreted as the amount of information contained in the message that converts the population share into the income share. The Theil index *T* is defined as:

(3) $$T=\sum_{i=1}^{N}\left(\frac{x_{i}}{\sum_{j=1}^{N}x_{j}}\cdot \ln\frac{x_{i}}{\bar{x}}\right)$$

where $x_{i}$ is a characteristic of agent *i*, $\bar{x}$ represents average income, and *N* is the population. The range of the Theil index is [0,∞). The larger is the value, the more obvious is the difference from the average.

## 3. Topology Control Game Model

In this section, a topology control game model is first constructed. Then, it is proved that the game model belongs to the ordinal potential game and the NE is Pareto optimal.

### 3.1. Utility Function

The environment of wireless sensor networks is relatively complex, and it is difficult to quantify the benefits of nodes. The existing topology control algorithm based on the ordinal potential game [15] does not adequately consider node revenue, and the utility function cannot accurately reflect the competition between nodes and the balance of energy consumption.

This paper uses a power control model based on the utility function [19]. To maximize utility function, each participant adjusts power in a selfish manner, which is typical for noncooperative power control games. In addition, to better balance the load between nodes, energy efficiency is improved. In this paper, the Theil index is introduced in the design of the utility function. In addition, the method of measuring income inequality in the field of social science is used to measure the imbalance of energy consumption between nodes in wireless sensor networks by using the node and its surplus energy as analogues for the group members and their income. Thus, a more accurate node utility function is obtained to describe the competitive relationship between nodes.

Consider a multihop network of independent and selfish nodes that adapt the transmit power levels according to their connectivity and energy consumption preferences. By considering the energy efficiency and energy balance, a specific utility function for node i(∀i∈N) is given by:

(4) $$u_{i}\left(p_{i},p_{-i}\right)=f_{p_{i}}\left(\lambda p_{i}^{max}-k_{p_{i}}p_{i}+\frac{1}{T+1}\right)+\frac{E_{r}\left(i\right)}{E_{0}\left(i\right)-E_{r}\left(i\right)}+\mu \bar{E_{i}\left(p_{i}\right)}$$

where, for node *i*, initial energy, current remaining energy, current transmitting power and maximum transmitting power are $E_{0}\left(i\right),E_{r}\left(i\right),p_{i}$ and $p_{i}^{max}$. $p_{-i}$ represents the transmit power of the remaining *n*-1 nodes except node *i*. In addition, we define a link state variable $f_{p_{i}}\left(f_{p_{i}}\geq 0\right)$. If the network is connected, then $f_{p_{i}}=1$; otherwise, $f_{p_{i}}=0$. Topology control aims to prolong the network lifetime by reducing the node power without destroying the overall network connectivity and robustness. By adding parameter $f_{p_{i}}\left(f_{p_{i}}\geq 0\right)$, it is ensured that the network remains connected after repeated iterations of the game. The $k_{p_{i}}$ represents the degree of node *i* when the transmitting power is $p_{i}$; λ and μ are the weight factors of the utility function and all are positive numbers. $\bar{E_{i}\left(p_{i}\right)}=\frac{1}{m}\sum_{j=1}^{m}\frac{E_{r}\left(j\right)}{E_{0}\left(j\right)-E_{r}\left(j\right)}$ (node *j* means that node *i* is a single hop neighbor node at power $p_{i}$, where *m* represents the number of one-hop neighbor nodes of node *i*) in Equation (4) indicates that more calls to the remaining high-energy nodes participate in the communication link to ensure load balancing [11]. To better balance the load between nodes and improve energy efficiency, the method of measuring income inequality in the field of social science is used to measure the imbalance of energy consumption between nodes in wireless sensor networks using the node and its residual energy as an analog for group members and their income; the Theil index *T* is defined as

$$T=\sum_{i=1}^{n}\left(\frac{E_{r}\left(i\right)}{\sum_{j=1}^{n}E_{r}\left(j\right)}\cdot \ln\frac{E_{r}\left(i\right)}{\bar{E_{r}}}\right).$$

The utility function satisfies the properties described in [22]. With the utility function defined, a game is played with all sensors picking their powers.

### 3.2. Model Proof

We show that the game $\Gamma =\langle N,S,\left\{u_{i}\right\}\rangle$ with the utility function of each sensor given by Equation (4) is an ordinal potential game; then, the existence of NEs is guaranteed.

**Theorem** **1.** *The game $\Gamma =\langle N,S,\left\{u_{i}\right\}\rangle$ is an ordinal potential game. The ordinal potential function is given by:*

(5) $$V\left(p_{i},p_{-i}\right)=\sum_{i\in N}^{}\left\{f_{p_{i}}\left(\lambda p_{i}^{max}-k_{p_{i}}p_{i}+\frac{1}{T+1}\right)+\frac{E_{r}\left(i\right)}{E_{0}\left(i\right)-E_{r}\left(i\right)}+\mu \bar{E_{i}\left(p_{i}\right)}\right\}$$

**Proof.**  We apply the asserted ordinal potential game in Equation (4). First, we have:

(6) $$\begin{array}{cc} \Delta u_{i} & =u_{i}\left(p_{i},p_{-i}\right)-u_{i}\left(q_{i},p_{-i}\right)\\ & =f_{p_{i}}\left(\lambda p_{i}^{max}-k_{p_{i}}p_{i}+\frac{1}{T+1}\right)+\mu \bar{E_{i}\left(p_{i}\right)}-f_{q_{i}}\left(\lambda p_{i}^{max}-k_{q_{i}}q_{i}+\frac{1}{T+1}\right)-\mu \bar{E_{i}\left(q_{i}\right)}\\ & =\left(f_{p_{i}}-f_{q_{i}}\right)\left(\lambda p_{i}^{max}+\frac{1}{T+1}\right)+f_{q_{i}}k_{q_{i}}q_{i}-f_{p_{i}}k_{p_{i}}p_{i}+\mu \left(\bar{E_{i}\left(p_{i}\right)}-\bar{E_{i}\left(q_{i}\right)}\right) \end{array}$$

Similarly,

(7) $$\begin{array}{cc} \Delta V & =V\left(p_{i},p_{-i}\right)-V\left(q_{i},p_{-i}\right)\\ & =\sum_{i\in N}^{}\left[f_{p_{i}}\left(\lambda p_{i}^{max}-k_{p_{i}}p_{i}+\frac{1}{T+1}+\mu \bar{E_{i}\left(p_{i}\right)}\right)\right]\\ & \;-\sum_{i\in N}^{}\left[f_{q_{i}}\left(\lambda p_{i}^{max}-k_{p_{i}}q_{i}+\frac{1}{T+1}+\mu \bar{E_{i}\left(q_{i}\right)}\right)\right]\\ & =\left(f_{p_{i}}-f_{q_{i}}\right)\left(\lambda p_{i}^{max}+\frac{1}{T+1}\right)+f_{q_{i}}k_{q_{i}}q_{i}-f_{p_{i}}k_{p_{i}}p_{i}+\mu \left(\bar{E_{i}\left(p_{i}\right)}-\bar{E_{i}\left(q_{i}\right)}\right)\\ & \;+\sum_{j\in N,j\neq i}^{}\left[\lambda \left(f_{p_{j}}-f_{q_{j}}\right)p_{j}^{max}+\mu \bar{E_{j}\left(p_{j}\right)}\right] \end{array}$$

Thus, we have:

(8) $$\Delta V=\Delta u_{i}+\sum_{j\in N,j\neq i}^{}\left[\lambda \left(f_{p_{j}}-f_{q_{j}}\right)p_{j}^{max}+\mu \bar{E_{j}\left(p_{j}\right)}\right]$$

For node *i*, because $f_{p_{i}}$ is monotonic and $\lambda \left(f_{p_{j}}-f_{q_{j}}\right)p_{j}^{max}+\mu \bar{E_{j}\left(p_{j}\right)}\geq 0$, it follows from Equation (6) that:

(9) $$\Delta u_{i}=\left\{\begin{array}{c} \geq 0\;if\;p_{i}>q_{i}\;and\;f_{p_{i}}>f_{q_{i}}\\ \leq 0\;if\;p_{i}<q_{i}\;and\;f_{p_{i}}<f_{q_{i}}\\ >0\;if\;p_{i}>q_{i}\;and\;f_{p_{i}}=f_{q_{i}}\\ <0\;if\;p_{i}>q_{i}\;and\;f_{p_{i}}=f_{q_{i}} \end{array}\right.$$

Therefore, $sgn\left(\Delta u_{i}\right)=sgn\left(\Delta V\right)$, the function $V(p_{i},p_{-i})$ is the ordinal potential function of the strategy game, and the strategy game $\Gamma =\langle N,S,\left\{u_{i}\right\}\rangle$ is the ordinal game. □

**Theorem** **2.** *The NE of the topology game model established in this paper is Pareto optimal [18] if the network $G_{max}$ is connected.*

**Proof.**  Due to the limited number of nodes, the number of nodes’ selectable transmit power sets is limited. According to [19], the finite ordinal potential game must converge to the NE (a strategy profile is a Nash equilibrium if no player can do better by unilaterally changing his or her strategy). According to the definition of the network model and the description of the utility function, a node maximizes the utility function by adjusting its own policy choice. The concrete manifestation is that the node continuously reduces the transmitting power and prolongs the survival time until the power of all nodes no longer changes; that is, the NE state is reached.

When a NE point is reached, if a node reduces its power, network connectivity will be destroyed. As a result, the remaining nodes must increase their power, thus resulting in lower utility function values of other nodes and disruption of the NE. Therefore, according to the Pareto optimal definition (in short, any form of resource reconfiguration is unlikely to benefit at least one person without harming anyone else), it can be concluded that the NE of the topological control game is Pareto optimal. □

## 4. Energy-Balanced Topology Control Game Algorithm

In this section, we propose an energy-balanced topology control algorithm in which each sensor adaptively adjusts its transmit power according to the residual energy, and the implementation process of each phase of the algorithm.

The node must meet certain preconditions when running the algorithm: (1) the network node ID must be unique; (2) the node cannot be moved after deployment; (3) the transmit power of all nodes is continuously adjustable; (4) the nodes in the network can get some information needed to perform the power game, such as: the average residual energy of the nodes in the network and the information of network connectivity [23]; and (5) when all nodes have the maximum transmit power characteristics, the network topology graph $G_{max}$ formed by default is connected (this must support bidirectional link communications).

Nodes in EBTG initiate with the maximum power network $G_{max}$ and then try to update this topology iteratively according to their increasing unwillingness. EBTG consists of three phases: the initialization phase (topological establishment phase), the adaptation phase (power adjustment phase), and the topology maintenance phase.

### 4.1. Initialization Phase

Every node in topology control game algorithms that makes a topological decision needs to collect some network information. In EBTG, the information required by node *i* in the topology construction process is the local topology $G_{i}$, which is an induced subgraph of $G_{max}$. Every vertex of the directed graph corresponds to a node in WSN.

To obtain this decision information, node *i* initializes its transmit power with maximum power $p_{max}$ and discovers its neighbor nodes by broadcasting the “Hello” Message and collecting the responses provided by the receivers at $p_{max}$. The message contains information such as node ID and remaining energy. By receiving and returning the message, a series of information, such as ID, transmit power and residual energy of its neighbor nodes, is learned and the maximum reachable neighbor set $N_{max}\left(i\right)$ of node *i* and its maximum uplink set $L_{max}\left(i\right)$ is determined. By establishing these sets, the largest global network topology view $G_{max}$ can be learned, thereby establishing a basis for subsequent routing decisions.

The detailed steps for initialization phase can be described as follows.

- **Step 1** Each node broadcasts the “Hello Message” with its maximum transmission power and sets a timeout for waiting for the reply message.
- **Step 2** It collects the neighbor node’s message under the specified timeout and establishes its neighbor list.
- **Step 3** A triad game model, namely $G_{max}$, is firstly built for the network.

### 4.2. Adaptation Phase

Node *i* in the adaptation phase determines its transmit power according to its current residual energy $E_{r}\left(i\right)$, the current transmitting power and the topology-related information collected during the initialization phase. The procedure of power adaptation is shown in Algorithm 1.

**Algorithm 1** EBTG power adaptation.
**initialization**
1:node *i* broadcasts “hello” message at $p_{i}^{max}$, *k* is the number of neighbors of node *i*2:Determine the neighbor set $N_{max}\left(i\right)$3:Determine the optional power set $P_{i}$ for node *i*, descending sort4:Broadcast optional power set $P_{i}$
**Power adjustment**
1:$P_{i}=\left\{p_{1},p_{2},\cdots ,p_{k}\right\}$, descending sort2:while $p_{i}$ ensure that the game is Nash equilibrium3:  for i=1,i≤N,i++4:    choose power according to $p_{i}^{*}=\underset{p_{i}\in P_{i}}{\arg\max}u_{i}\left(p_{i},p_{-i}\right)$5:    if $u_{i}^{*}\left(p_{i}^{*},p_{-i}\right)\geq u_{i}\left(p_{i},p_{-i}\right)$6:      if $p_{i}$ ensure that the game is Nash equilibrium7:        $p_{i}=p_{i}^{*}$, update $p_{i}$8:      end if9:    end if10:  end for11:  broadcast a “hello” message including the new power setting $p_{i}$ at $p_{i}^{max}$12:end while


This paper adjusts the network topology structure through the power control method, sets the transmission power of the node as the optimal transmission power, and thus obtains the optimized network topological structure.

In the power adaptation phase, we first need to sort the node’s strategy set $P_{i}=\left\{p_{1},p_{2},\cdots ,p_{k}\right\}$ in descending order. The minimum available transmit power $p_{i}^{min}$ of node *i* can be calculated using the free-space model proposed in [24]. Algorithm 1 shows the pseudo-code of the adaptation phase.

**Figure 1:**
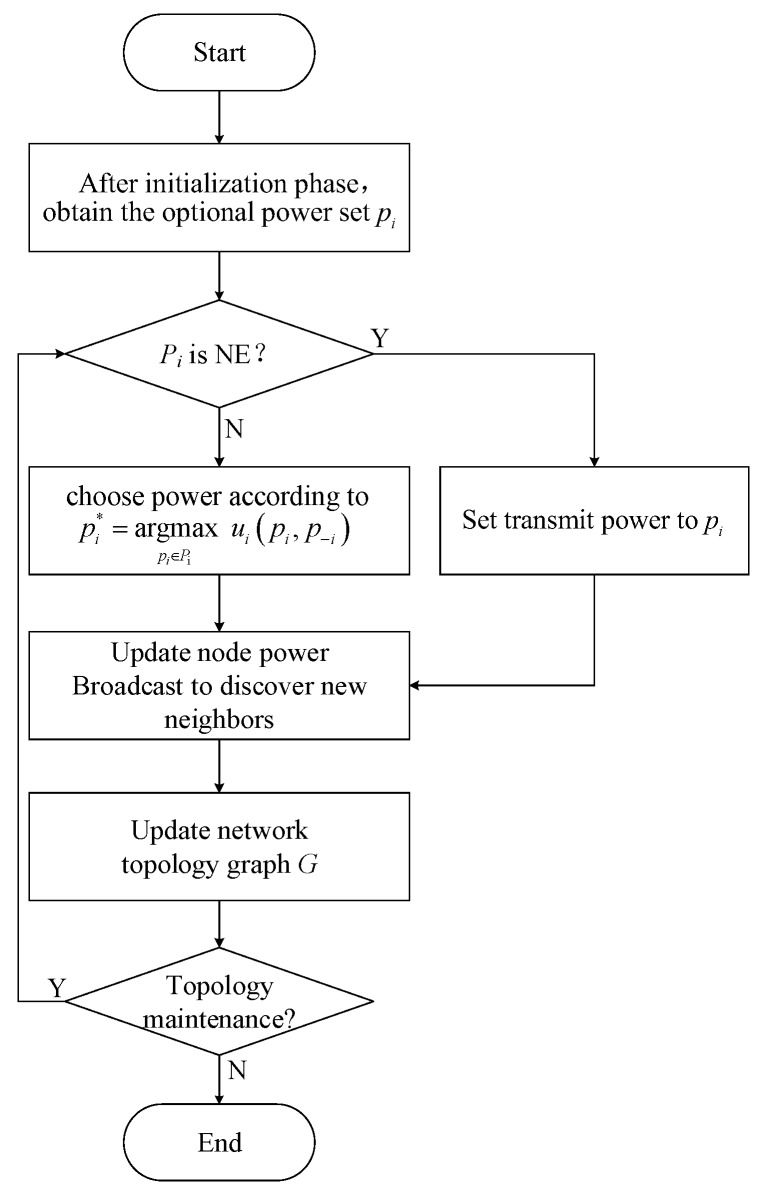
Flowchart of power adaptation.

The power adjustment sequence of the EBTG algorithm is based on the node ID, as shown in Figure 1, the transmit power of one node is adjusted for each round, and the remaining power of the node is unchanged. To ensure convergence to the NE, this algorithm uses the better response strategy update scheme proved in [19], which converges to the NE in the finite ordinal potential game. Given the power $p_{-i}$ of other participants, the optimal response of node *i* is $r_{i}\left(p_{-i}\right)=\min\left(p_{i}^{max},p_{i}^{*}\right)$, which has $p_{i}^{*}=\underset{p_{i}\in P_{i}}{\arg\max}u_{i}\left(p_{i},p_{-i}\right)$. During the game, when the node selects a power lower than the current transmit power for communication, it is observed whether the corresponding integrated utility function value increases. If it is larger, it indicates that the lower power is more suitable for use as the transmit power; otherwise, the node keeps current transmit power unchanged.

The detailed steps for adaptation phase can be described as follows.

- **Step 1** When the initialization phase is completed, the node has learned the current transmit power $p_{i}$, remaining energy $E_{r}\left(i\right)$, and optional power set $P_{i}=\left\{p_{1},p_{2},\cdots ,p_{k}\right\}$, prepare for the adaptation phase.
- **Step 2** Each node calculates its own utility function value according to Equation (4), and then broadcasts its calculation result of $u_{i}\left(p_{i},p_{-i}\right)$ within the timeout.
- **Step 3** A node receives the utility function value of its neighbor node and stores $u_{i}\left(p_{i},p_{-i}\right)$ to the corresponding node in the neighbor list. After getting all payoffs of nodes in the neighbor list, each node sorts the utility function value (includes itself) out in terms of descending order.
- **Step 4** According to the better response strategy, the transmit power with greatest $u_{i}\left(p_{i},p_{-i}\right)$ are selected as the optimal transmit power by the game from the network. Then, the node broadcasts the optimal transmit power which it has selected.

**Figure 2:**
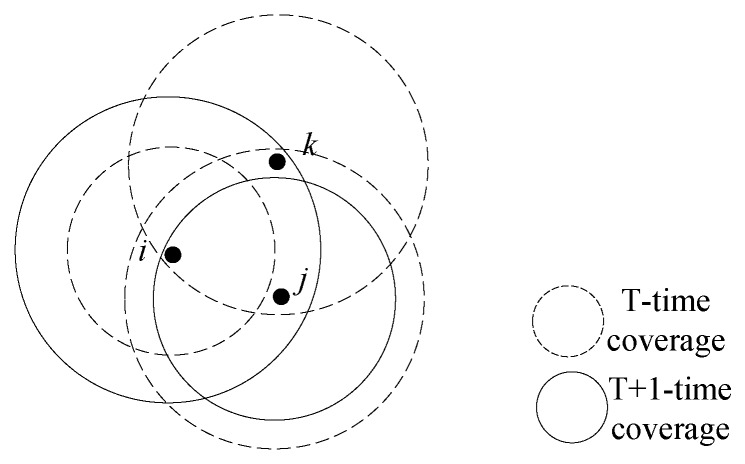
Diagram of power adaptation.

When the power of the node is changed, the communication radius, the neighboring node and its related links will change, which leads to the change of the network topology. As shown in Figure 2, when the transmit power of node *i* increases, node *j* will be included in its communication range; then, the nearest neighbor node of node *i* is changed from the original node *k* to the current node *j*. Therefore, node *j* can reduce its transmit power appropriately under the precondition of guaranteeing full network connectivity.

### 4.3. Topology Maintenance Phase

As time flows, the energy consumption of the nodes may become more unbalanced. Network topology maintenance must be performed dynamically, considering that the energy consumption between nodes will become unbalanced and possible node failures or deaths. For the topology maintenance phase, we designed an event-triggered approach that adaptively regenerates a more balanced network topology.

The power game process can be implemented by comparing the residual energy of nodes with the energy threshold or by setting the period to balance the load of nodes and prolong the network lifetime. The detailed steps for topology maintenance phase can be described as follows. Algorithm 2 shows the pseudo-code of the topology maintenance phase.

**Algorithm 2** EBTG topology maintenance.
**initialization**
1:Receive neighbor information2: Compare with energy threshold3:  Replay the game of power adaptation

- **Step 1** When the reduction in the energy level of node *i* exceeds a given threshold, the topology maintenance request message is broadcast using the maximum power $p_{i}^{max}$ and the neighbor table is updated with the messages returned by each neighbor node. Any neighbor node that does not return the message is marked as dead.
- **Step 2** Node *i* replays the game according to the latest information obtained and selects the power $p_{i}^{*}$ that obtains the maximum utility value in the current state as its transmission power and determines the connectivity.
- **Step 3** Determine whether the network is connected [23].
- **Step 4** When the network is in the connected state, the node can be used for communication; and, when the network is in an unconnected state, the node needs to clear the local neighbor table and broadcast the topology reconstruction message at the maximum power $p_{i}^{max}$. When the surviving nodes in the network receive the message, they enter the topology maintenance phase (they re-execute the initialization phase).

**Theorem** **3.** *If the $G_{max}$ is a connected network, the EBTG algorithm converges to the NE state that can maintain the connectivity of network $G_{max}$.*

**Proof.**  It is known from Theorem 1 that the topological control game model constructed in this paper is an ordinal potential game. In the EBTG algorithm proposed in this paper, the node increases its benefit function value by adjusting the choice of strategy (i.e., reducing the power value of the node) until the selection strategies of all nodes are not changed. Obviously, this state is a NE. It is assumed that node *i* obtains greater benefits in the power $p_{i}<p_{i}^{*}$, and the network is disconnected when the power $p_{-i}$ of the other nodes is unchanged; therefore:

(10) $$u_{i}\left(p_{i},p_{-i}\right)=\frac{E_{r}\left(i\right)}{E_{0}\left(i\right)-E_{r}\left(i\right)}+\mu \bar{E_{i}\left(p_{i}\right)}>\lambda p_{i}^{max}-k_{p_{i}}p_{i}^{*}+\frac{E_{r}\left(i\right)}{E_{0}\left(i\right)-E_{r}\left(i\right)}+\mu \bar{E_{i}\left(p_{i}^{*}\right)}$$

In addition,

(11) $$\mu \bar{E_{i}\left(p_{i}\right)}>\lambda p_{i}^{max}-k_{p_{i}}p_{i}^{*}+\mu \bar{E_{i}\left(p_{i}^{*}\right)}$$

Obviously, Equation (10) is not tenable, thus obtaining the connected network at each round of the game execution of the EBTG algorithm. □

## 5. Simulation Results Analysis

**Figure 3:**
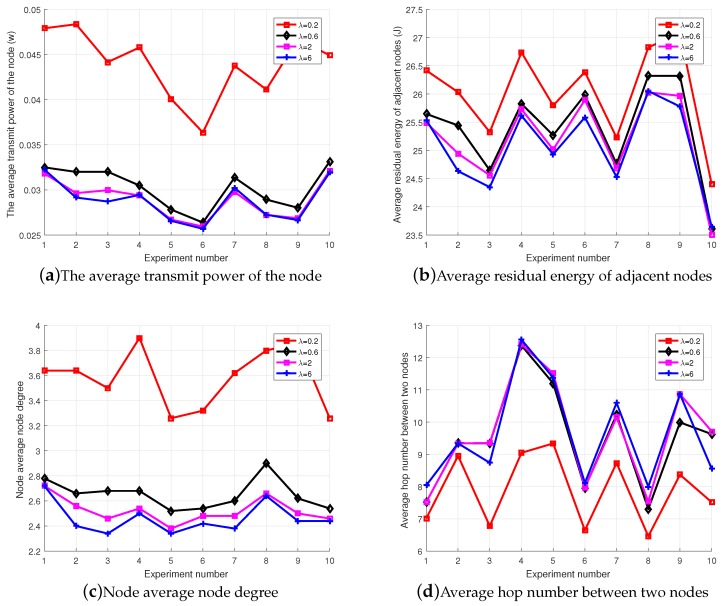
The impact of λ on network performance.

In this section, results of computer simulations are provided to illustrate the proposed algorithms. MATLAB R2016a was used as a simulation tool to simulate the EBTG algorithm. In addition, a comparison with the DIA [10], MLPT [11] and DEBA [15] algorithms was conducted with regard to node degree, node transmit power, node hop number and node residual energy. The experiment assumed that all nodes are randomly deployed and cannot be moved. Since this paper only involves the design and implementation of the topology control algorithm and real traffic information which depends on applications is varied, we used this assumption instead of real application traffic. Furthermore, due to a high volume of control messages (e.g., route discovery information) flooded through the whole WSN, the control traffic is in contention mode and most of this control traffic is broadcast, most works in this field assume the same average rate of traffic between every pair of nodes [25,26], thus we made the following assumptions: each sensor sends a packet to other sensors per second, i.e., each sensor transmits n−1 packets per second; the packet size is 1024 bytes; and the transmission rate is $10^{6}$ bits/s. The remaining emulation parameters are shown in Table 1:

First, weight factor λ and μ in the utility function must be determined; the experiment randomly distributed 50 nodes in the target region, as shown in Figure 3.

For μ=1, the influence of λ on the network topology performance was considered in terms of the average transmit power, the average node degree between nodes, the average residual energy of the adjacent node and the average hop number of the shortest path between nodes.

Figure 3a indicates that the average transmit power of the node decreases as λ increases. Figure 3b indicates that the residual energy of the neighboring node decreases as λ increases. Figure 3c indicates that the average node degree of the network decreases as λ increases, but tends to stabilize after λ≥2. Figure 3d indicates that the average hop number of the shortest path between nodes increases as λ increases. The changes after λ≥2 also tended to stabilize. From the general theory of network topology, it can be seen that the topology of the network is perfect when the transmit power of the nodes is low, while there is a moderate node degree and average hop number. By comprehensively considering node computing power and network performance [27], when λ≥2, the impact on network performance has become similar, thus, for the convenience of calculation, the value of λ was taken as the intermediate value of 2 and 6, and the value of μ was taken as 1.

**Figure 4:**
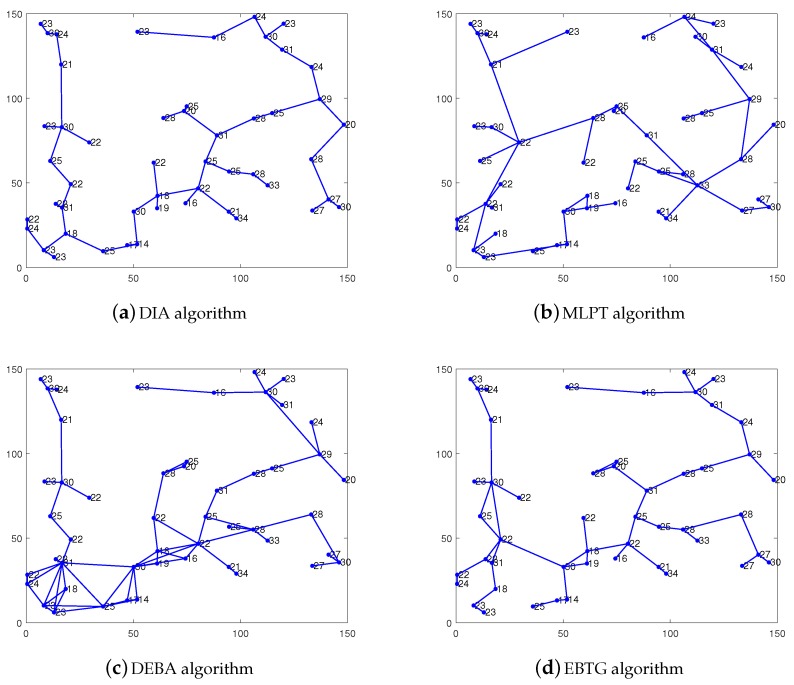
Network topology comparison chart.

Figure 4 shows the network topology diagram of the four algorithms, i.e., DIA, MLPT, DEBA and EBTG. It can be seen that the network topology built by the DIA algorithm has a large load and low residual energy (the nodes are marked out). The DEBA algorithm has a higher node degree and more redundant nodes, which lead to faster energy consumption. Compared to the other two algorithms, the MLPT and EBTG algorithms have lower node degrees and fewer redundant nodes. The general theory of network topology shows that the EBTG algorithm has moderate nodes and redundant nodes; therefore, its network connectivity and robustness are better than those of the other three algorithms, which can efficiently balance the load between nodes to prolong network lifetime.

To make a clearer comparison of the four algorithms, eight groups of experiments were conducted. The specific experimental parameters are set as shown in Table 1, where the number of nodes participating in the experiment was increased from 30 to 100 and the algorithms were compared by calculating their node transmit power, the hops of the shortest link between nodes and the average value of the node degree.

**Figure 5:**
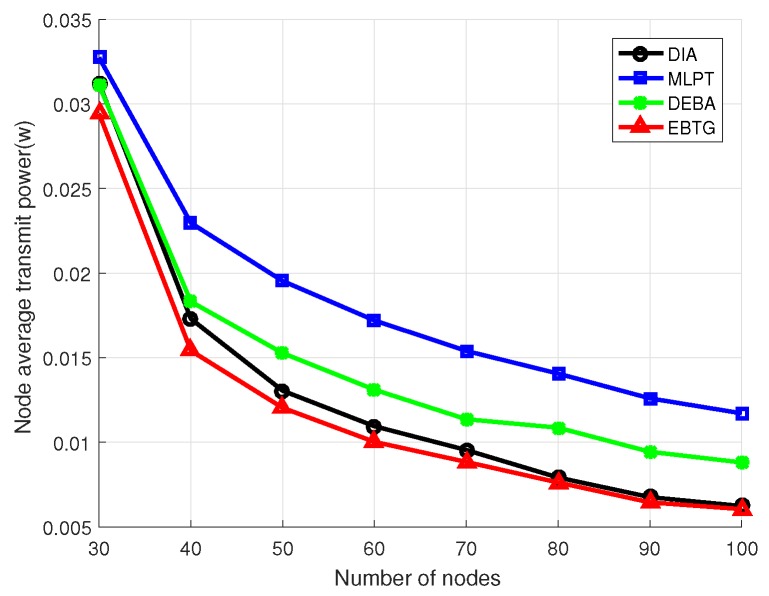
Node average transmit power.

Figure 5 is a comparison diagram of the transmission power between nodes. It can be observed in the figure that the transmission power of a node decreases as the number of nodes increases. The EBTG algorithm’s node average transmit power is lower than those of DIA, MLPT and DEBA algorithms, which can ensure that the EBTG algorithm can establish network topology connections with lower power, which is conducive to extending the network lifetime. In the design stage of EBTG algorithm, we considered the influence of node transmit power on the operation of the algorithm. Therefore, the simulation results show that the node transmit power performance is optimal.

**Figure 6:**
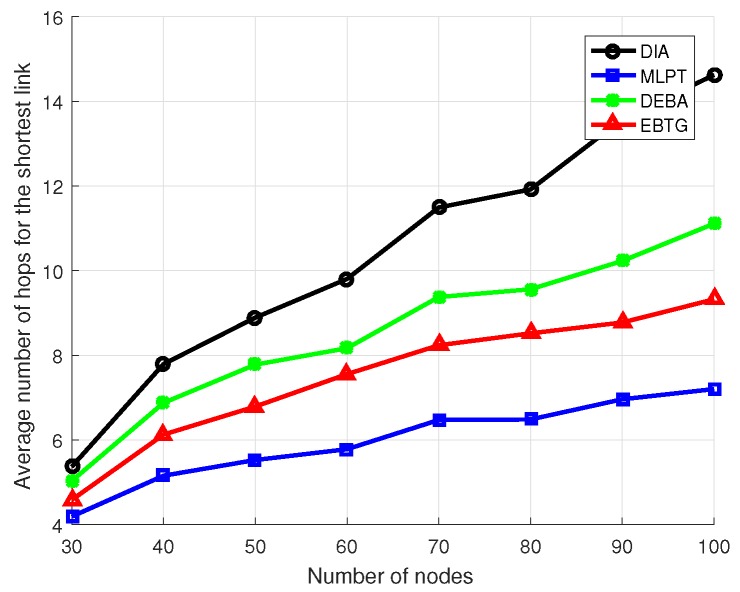
Average number of hops for the shortest link.

Figure 6 shows the hop count comparison of the shortest link between nodes. The average hop count of the EBTG algorithm is higher than that of the MLPT algorithm, but it is still lower than the DIA and DEBA algorithms. The MLPT algorithm has higher node transmit power and greater communication coverage, and thus its average link hop count is lower. Since the EBTG algorithm operates at lower power and the communication radius is smaller, the average hop count of the link increases. However, the EBTG algorithm still obtains fewer link hops than the DIA and DEBA algorithms when the transmit power is lower than the DIA algorithm.

**Figure 7:**
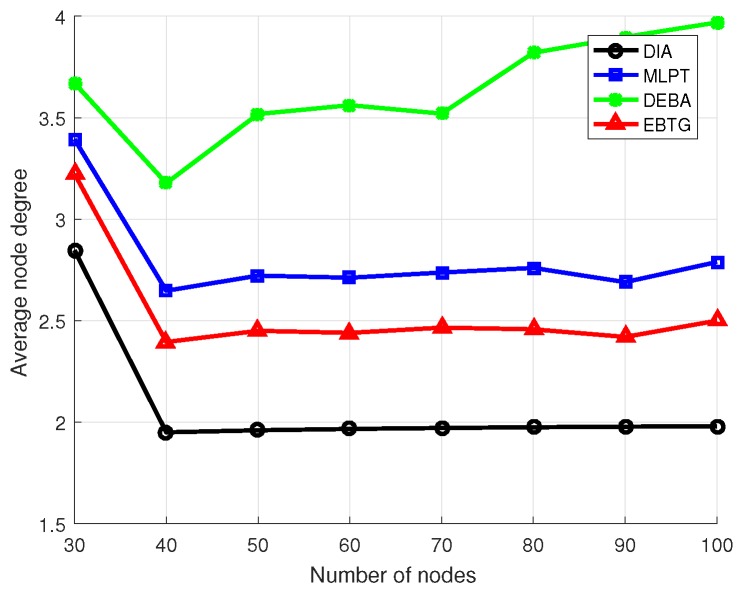
Average node degree.

Figure 7 shows a comparison of node degrees for the four algorithms. Because nodes with more energy remaining in the EBTG algorithm are more active in node communication, to obtain a more balanced load to prolong the life cycle of the network, the node degree is higher than that of the DIA algorithm but lower than that of the DEBA and MLPT algorithms. The moderate node degree of the EBTG algorithm does not occupy too much of the energy resources and obtains relatively good connectivity and robustness, while having fewer redundant nodes can achieve better energy efficiency, improve channel multiplexing and reduce interference. Since the influence of network node degree on network performance, such as robustness. is obvious, we considered the influence of node degree when designing the algorithm’s utility function. It can be seen in the figure that the node degree of the EBTG algorithm performs better than those of the other algorithms, which do not effectively consider the influence of node degrees.

**Figure 8:**
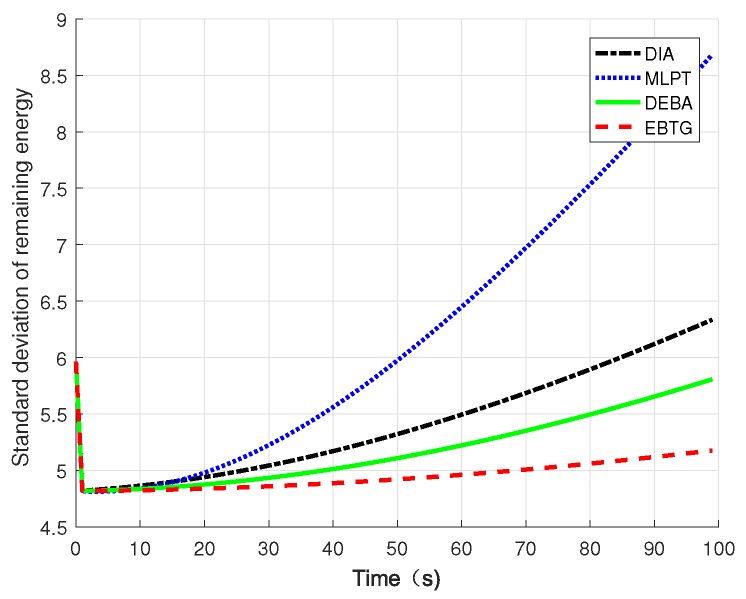
Standard deviation of node residual energy.

Figure 8 compares the standard deviations of the node residual energy. The residual energy standard deviation was calculated using the formula $\sigma_{E_{r}}=\sqrt{\frac{1}{N}\sum_{i}^{N}{\left(E_{r}\left(i\right)-\bar{E_{r}}\right)}^{2}}$ in the simulation to compare the remaining energy balance. It can be seen that the variance of the EBTG algorithm changes slowly. In the network topology constructed by the DIA, MLPT, and DEBA algorithms, the load of some nodes is too high, which affects the network lifetime. If these heavily loaded nodes die prematurely, they will also have a greater impact on the connectivity and robustness of the network. The DIA algorithm overemphasizes that reducing the node transmit power makes the network energy consumption uneven; the MLPT algorithm does not consider the node’s residual energy, resulting in poor performance of its energy balance; the DEBA algorithm focuses on energy balance, while ignoring the energy efficiency, which leads to the growth of the residual energy standard deviation; the rising trend of the EBTG algorithm is the most gradual. The EBTG algorithm not only considers the remaining energy of the node but also transfers the data forwarding task to nodes with more residual energy, effectively balancing the load of the entire network and improving energy efficiency.

**Figure 9:**
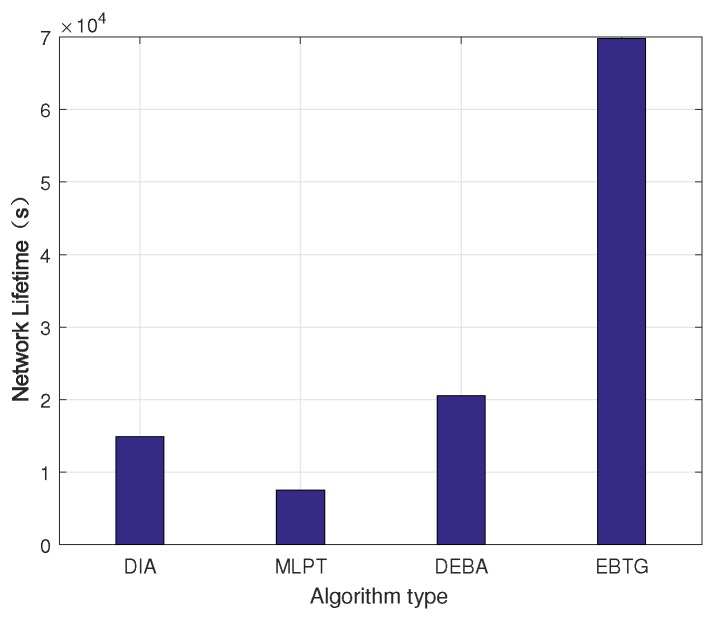
Network lifetime.

Figure 9 is a network lifetime comparison chart. Because topology control is mainly concerned with energy, prolonging the network life cycle is an important index for evaluating the topology control algorithm. The graph shows that the network lifetime of the EBTG algorithm is the longest because the EBTG algorithm reduces the transmit power of the node, expertly balances the load between nodes and improves the energy efficiency; therefore, its network lifetime is much higher than those of the networks constructed using the DIA, MLPT and DEBA algorithms.

## 6. Conclusions

Sensors in wireless sensor networks have been restricted to local communications and want to reduce their own energy consumption selfishly. Furthermore, the unbalanced energy consumption between nodes is likely to shorten the network lifetime.

Based on the theory of potential games and the Theil index, an optimized utility function was proposed for WSNs. This function takes the residual energy of nodes, the transmitting power of nodes and the connectivity of the network into account. On this basis, a topological game model was constructed, and we proved that there is Pareto optimal NE in the model. Thus, an energy-balanced WSN distributed topology game algorithm called EBTG was proposed. From the simulation results, it can be concluded that the EBTG algorithm can effectively reduce the power of the transmitting node, balance the load between nodes, improve the energy efficiency of the network and prolong the network lifetime to ensure network connectivity and robustness.

In future work, we will study the operation of this algorithm in a real-world wireless communication environment to improve the reliability and stability of the algorithm.

## Figures and Tables

**Table 1:** Experimental parameters.

Parameter Name	Parameter Size
Monitoring area	150 m × 150 m
Communication radius	50 m
Node initial energy	50 J
Wavelength λ	0.1224 m
Receiving threshold	7 × 10${}^{-10}$ w
Transmit antenna gain $G_{t}$	1
Receive antenna gain $G_{r}$	1
System loss *L*	1
